# Investigation of Bone Growth in Additive-Manufactured Pedicle Screw Implant by Using Ti-6Al-4V and Bioactive Glass Powder Composite

**DOI:** 10.3390/ijms21207438

**Published:** 2020-10-09

**Authors:** Tu-Ngoc Lam, Minh-Giam Trinh, Chih-Chieh Huang, Pei-Ching Kung, Wei-Chin Huang, Wei Chang, Lia Amalia, Hsu-Hsuan Chin, Nien-Ti Tsou, Shao-Ju Shih, San-Yuan Chen, Chun-Chieh Wang, Pei-I Tsai, Meng-Huang Wu, E-Wen Huang

**Affiliations:** 1 Department of Materials Science and Engineering, National Chiao Tung University, 1001 University Road, Hsinchu 30010, Taiwan; lamtungoc1310@gmail.com (T.-N.L.); Sigher@itri.org.tw (C.-C.H.); peggygong1014@gmail.com (P.-C.K.); jason910140@yahoo.com.tw (W.C.); lia.amalia.m55@gmail.com (L.A.); refryan3169@gmail.com (H.-H.C.); tsounienti@nctu.edu.tw (N.-T.T.); sanyuanchen@mail.nctu.edu.tw (S.-Y.C.); 2 Department of Physics, College of Education, Can Tho University, Can Tho City 900000, Vietnam; 3 International Ph.D. Program in Medicine, College of Medicine, Taipei Medical University, No. 250, Wuxing St, Xinyi District, Taipei 11031, Taiwan; minhgiam1310@gmail.com; 4 Department of Trauma-Orthopaedics, College of Medicine, Pham Ngoc Thach Medical University, No. 02, Duong Quang Trung St, 10th District, Ho Chi Minh City 700000, Vietnam; 5 Laser and Additive Manufacturing Technology Center, Industrial Technology Research Institute, Hsinchu 31040, Taiwan; Horus-Huang@itri.org.tw; 6 Department of Materials Science and Engineering, National Taiwan University of Science and Technology, No. 43, Sec. 4, Keelung Road, Taipei 10607, Taiwan; shao-ju.shih@mail.ntust.edu.tw; 7 National Synchrotron Radiation Research Center, Hsinchu 30076, Taiwan; wang.jay@nsrrc.org.tw; 8 Biomedical Technology and Device Research Laboratories, Industrial Technology Research Institute, Hsinchu 31040, Taiwan; peiyi@itri.org.tw; 9 Department of Orthopaedics, School of Medicine, College of Medicine, Taipei Medical University, No. 250, Wuxing St, Xinyi District, Taipei 11031, Taiwan; 10 Department of Orthopedics, Taipei Medical University Hospital, No. 252, Wuxing St, Xinyi District, Taipei 11031, Taiwan

**Keywords:** pedicle screw implant, additive manufacturing, bioactive glass, osseointegration, stress-shielding effect, X-ray tomography

## Abstract

In this study, we optimized the geometry and composition of additive-manufactured pedicle screws. Metal powders of titanium-aluminum-vanadium (Ti-6Al-4V) were mixed with reactive glass-ceramic biomaterials of bioactive glass (BG) powders. To optimize the geometry of pedicle screws, we applied a novel numerical approach to proposing the optimal shape of the healing chamber to promote biological healing. We examined the geometry and composition effects of pedicle screw implants on the interfacial autologous bone attachment and bone graft incorporation through in vivo studies. The addition of an optimal amount of BG to Ti-6Al-4V leads to a lower elastic modulus of the ceramic-metal composite material, effectively reducing the stress-shielding effects. Pedicle screw implants with optimal shape design and made of the composite material of Ti-6Al-4V doped with BG fabricated through additive manufacturing exhibit greater osseointegration and a more rapid bone volume fraction during the fracture healing process 120 days after implantation, per in vivo studies.

## 1. Introduction

As the most indispensable treatment of serious spinal disorders and deformities, the pedicle screw instrumentation, first described by Boucher [1] and reintroduced by Roy-Camille [2], has been widely used for rigid spinal fixation and stabilization in spinal surgery [3,4,5,6,7,8,9,10]. However, the breakage and loosening of pedicle screws are the major clinical problems that drastically affect the outcomes of spinal surgery [5,8,11,12]. Significant approaches have been proposed for screw design modification and screw surface treatment to obtain better mechanical binding at the bone–screw interface through greater bone ingrowth and osseointegration [3,4,6,8,13,14]. Nevertheless, more efforts are required to optimize the shape and material composition of pedicle screws to increase their efficacy in various biological applications [15].

Based on the bone remodeling mechanism proposed by Wolff’s law [16], several efforts have been undertaken to design healing chambers in terms of the shape of the threads, thread pitch [17,18], and surgical drilling dimensions of screw implants [19,20]. In the authors’ previous work [21], a novel numerical method was proposed to optimize the shape of the healing chamber of titanium–aluminum–vanadium alloy (Ti-6Al-4V) screw implants under any condition to maximize the volume of the healthy surrounding bone. Such an effective algorithm was adopted in the current work, wherein the model of spinal implant and the corresponding loading conditions were considered. This approach could help derive an optimal design of the healing chamber in pedicle screws fabricated using additive manufacturing that could promote biological healing. The efficacy of this design was examined through in vivo experiments.

Titanium (Ti)-based alloys have been employed in medical applications because of their biocompatibility in the human body. Notably, Ti-6Al-4V is an essential component of biomedical implants [22], and its mechanical properties have been determined to be well adapted to bone growth [23] when examined [24,25]. Hence, additive-manufactured Ti-6Al-4V implants have garnered attention [26,27] as biocompatible materials. 

Ti-6Al-4V alloys are favorable implants for high load bearings in clinical applications because they possess superior mechanical strength and exhibit high biomechanical performance combined with a rapid bone remodeling process, per in vivo studies [28,29,30,31]. Upon exploring new bone-like materials that have stiffness similar to the surrounding bone tissue to minimize the stress-shielding effects and enrich osseointegration [32], bioactive glasses (BGs), first discovered by Hench et al. [33], have attracted significant attention recently for their use in various medical applications, such as bone implants [34], dentistry [35,36], and drug delivery [37,38] because of their high reactive surface areas, excellent biocompatibility, and bioactivity [34]. BGs were observed to firmly anchor with the living tissues through the formation of hydroxyapatite (HA) minerals, which are the major components of mineralized mature bone responsible for enduring high loads [7,8]. Although comprehensive research has been devoted to new composite materials of metals coupled with BG-based implants in attaining sufficient strength and rich osseointegration, details regarding their in vivo performance are still lacking and must be studied for clinical applications.

Based on the authors’ earlier study, the BG composition of 58S exhibited a higher growth rate of HA and better bioactivity [39]. Therefore, we were motivated to conduct further extensive research on bone incorporation and osseointegration performance of pedicle screw implants comprising Ti-6Al-4V doped with BG composite material with optimal shape design of the healing chamber through in vivo studies. Moreover, whether the addition of 58S BG to Ti-6Al-4V or the application of the novel numerical model-based optimal healing chamber in pedicle screws fabricated using three-dimensional (3D) printing influences bone regeneration process in vivo needed to be determined. In addition, the in vivo osseointegration performance of 3D-printed pedicle screw implants comprising Ti-6Al-4V doped with 58S BG composite with an optimal shape design must be assessed.

In the present study, we investigated the effects of the geometry and composition of three types of pedicle screw implants fabricated using the selective laser melting (SLM) process and compared them with the commercially available Ti-6Al-4V pedicle screw implants in terms of the degree of osseointegration and bone remodeling in a porcine model. Four types of pedicle screw implants with commercial and optimal designs composed of Ti-6Al-4V and Ti-6Al-4V doped with BG were prepared, namely the commercial Ti-6Al-4V, optimal Ti-6Al-4V, commercial Ti-6Al-4V doped with BG, and optimal Ti-6Al-4V doped with BG. Imaging techniques, such as histological staining, fluorescence, X-ray micro-computed tomography (µCT), and transmission X-ray microscopy (TXM) analyses, were systematically conducted to evaluate the development of bone integration with the four types of pedicle screws during the same implantation period.

## 2. Results

### 2.1. Reduced Stress-Shielding Effect through BG Doping

**Figure 1:**
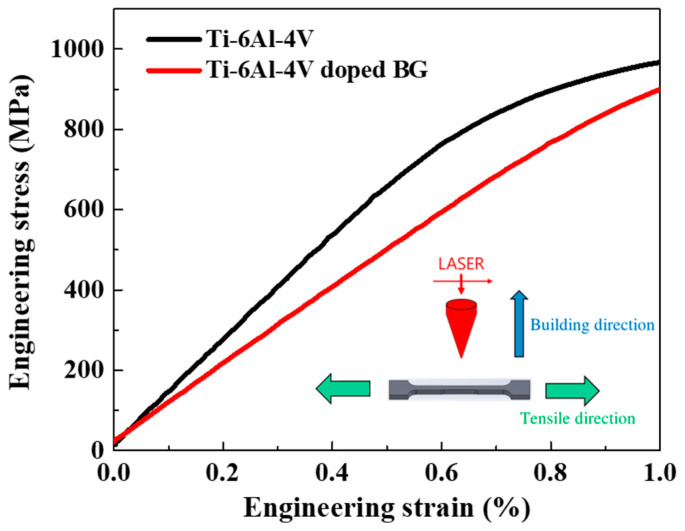
Macroscopic engineering stress–strain (S–S) curves in the elastic regimes of titanium-aluminum-vanadium (Ti-6Al-4V) and Ti-6Al-4V doped with bioactive glass (BG) samples. The schematic of tensile dog-bone specimens is presented in the inset.

Figure 1 reveals the uniaxial tensile engineering stress–strain (S–S) curves of 3D-printed samples of Ti-6Al-4V and Ti-6Al-4V doped with BG. The elastic modulus of 3D-printed Ti-6Al-4V doped with BG was 93 GPa, which was 28% lower than that of 3D-printed Ti-6Al-4V (129 GPa), implying superior adaptability of Ti-6Al-4V doped with BG to the bone. Notably, doping with BG effectively reduced the elastic modulus mismatch between Ti-6Al-4V doped with BG (93 GPa) and the bone (10–30 GPa) [40], thereby minimizing the stress-shielding effects. This crucial role of BG is expected to be beneficial in the bone remodeling process and bone healing during the implantation period.

### 2.2. Highest Bone Volume Fraction in the Optimal Ti-6Al-4V Doped with BG

**Figure 2:**
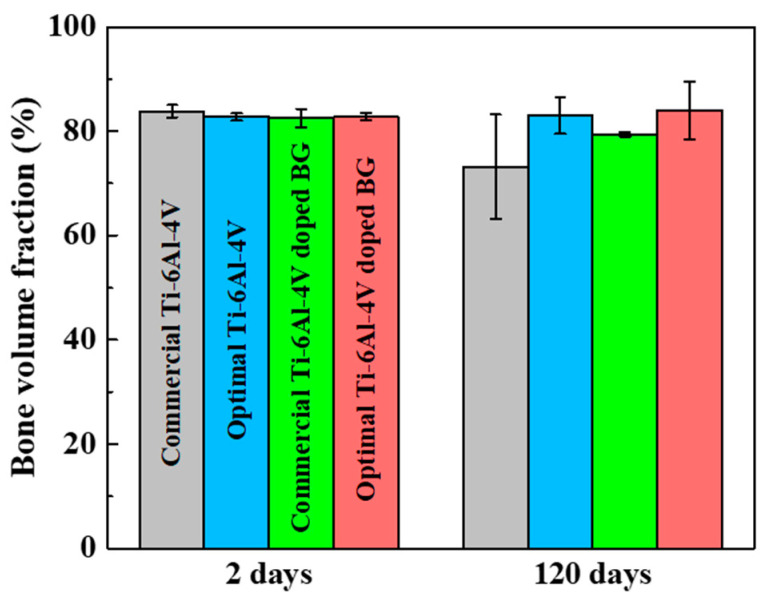
Bone volume fraction of the commercial Ti-6Al-4V, optimal Ti-6Al-4V, commercial Ti-6Al-4V doped with BG, and optimal Ti-6Al-4V doped with BG pedicle screws at 2 days and 120 days after implantation.

Through µCT, we investigated the bone volume fraction of healthy surrounding bone around the four types of pedicle screws at 2 and 120 days after implantation, as displayed in Figure 2. The bone volume fractions with the commercial Ti-6Al-4V, optimal Ti-6Al-4V, commercial Ti-6Al-4V doped with BG, and optimal Ti-6Al-4V doped with BG implants after two days of implantation were calculated to be 83.8%, 82.7%, 82.5%, and 82.7%, respectively, with a negligible difference in bone volume fraction among the four implants. The adaptation effectiveness of the four pedicle screws was assessed using their corresponding bone volume fractions during the same implantation period. A significant difference was observed among the four pedicle screw implants after 120 days in terms of the development of the surrounding bone. Notably, the commercial Ti-6Al-4V had the lowest bone volume fraction of 73.2%, with the slowest growth of healthy surrounding bone. Higher bone volume fractions with the other three pedicle screws imply that either doping with BG or applying the optimal shape design accelerates healthy bone growth with the use of the 3D print–based pedicle screw implants. Moreover, among implants made of the same composite material of Ti-6Al-4V or Ti-6Al-4V doped with BG, the optimal shape design evidently enhanced the bone volume fraction from 73.2 or 79.4% to 83.0% or 84.0%, probably because of the greater bone ingrowth into the optimal design of the healing chamber. Doping with BG increased the bone volume fraction of implants owning the same commercial shape design from 73.2% to 79.4%, however, there was a negligible discrepancy between pedicle screws owning the same optimal shape design composed of Ti-6Al-4V (83.0%) or Ti-6Al-4V doped with BG (84.0%). Our results suggested that the geometric effect rather than the composition effect is more beneficial for the bone volume fraction. The combined effects of geometry and composition led to the highest bone volume fraction in the pedicle screw of optimal Ti-6Al-4V doped with BG, suggesting the most rapid bone growth process in this promising implant.

### 2.3. Reliable TXM Imaging Technique for Bone Characterization

In addition to the volume fraction of healthy surrounding bone, the developing status of immature bone and mature bone at the intact bone–implant interface is crucial in evaluating the maximum feasible success of the pedicle screw implants in practical orthopedic applications. We performed synchrotron-based TXM with a high spatial resolution to observe the interfacial contact of immature bone, mature bone, and void regions with the pedicle screws at a microstructural scale. Bone characterization analyzed using TXM was compared with that analyzed using traditional imaging techniques.

**Figure 3:**
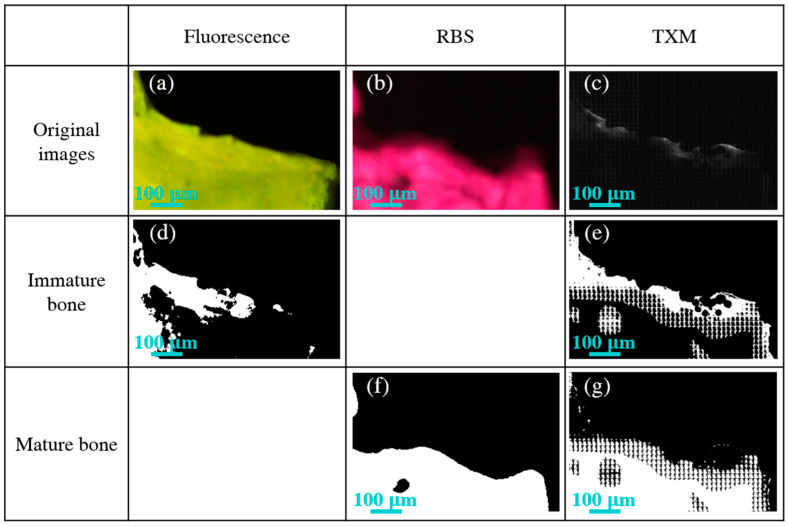
Microscopic images of healthy surrounding bone in the commercial Ti-6Al-4V doped with BG pedicle screw obtained using (**a**) fluorescence, (**b**) Sanderson’s Rapid Bone Stain (RBS), and (**c**) transmission X-ray microscopy (TXM) imaging modalities. (**d**) The white zones of immature bone seen with fluorescence. (**f**) The white areas of mature bone obtained using RBS. The white regions of immature bone (**e**) and mature bone (**g**) observed using TXM.

Figure 3a–c present the microscopic images obtained using fluorescence, RBS, and TXM imaging techniques of the bone formed around the commercial Ti-6Al-4V doped with BG pedicle screw 120 days after implantation. The brighter green area in the fluorescence image represents only immature bone, and the pink-stained zone exhibits only mature bone. By contrast, TXM enables the differentiation between immature bone and mature bone through image contrast in grayscale color. The areas of immature bone analyzed using fluorescence (32.6%) in Figure 3d and TXM (32.0%) in Figure 3e were well matched. In addition, an excellent correlation was obtained regarding the mature bone analyzed using RBS (70.7%) in Figure 3f and TXM (67.7%) in Figure 3g. Hence, in addition to traditional imaging techniques, the reliability of image analysis with TXM is possibly applicable to specifying the status of bone remodeling at the bone–implant interface during the fracture healing process. 

### 2.4. Rapid Bone Graft Incorporation in Optimal Ti-6Al-4V Doped with BG

**Figure 4:**
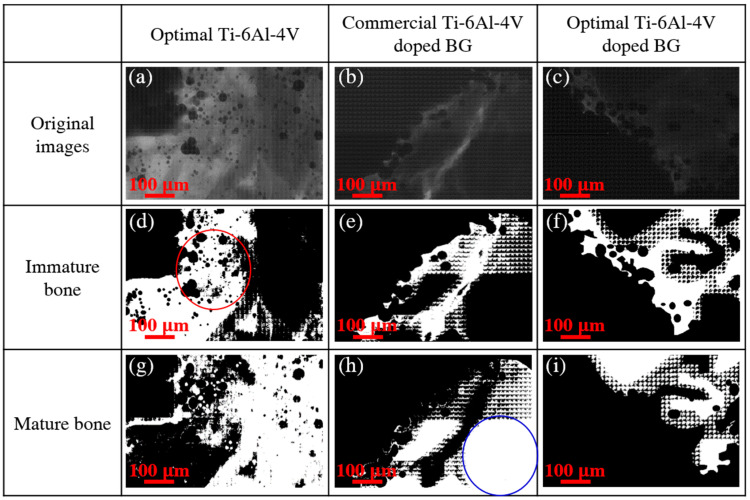
2D TXM images of bone growth around the three 3D-printed pedicle screw implants of (**a**) optimal Ti-6Al-4V, (**b**) commercial Ti-6Al-4V doped with BG, and (**c**) optimal Ti-6Al-4V doped with BG 2 days after implantation. The white regions of immature bone and mature bone of optimal Ti-6Al-4V are illustrated in (**d**) and (**g**); those of the commercial Ti-6Al-4V doped with BG in (**e**,**h**); and those of the optimal Ti-6Al-4V doped with BG in (**f**) and (**i**). The red and blue marked circles represent the unconnected and connected distributions, respectively.

We further conducted TXM to identify the bone remodeling status of immature bone, mature bone, and void around the three 3D-printed pedicle screws during the same implantation period. Figure 4 displays the 2D TXM images of direct osseous apposition with pedicle screws in the 3D printing of optimal Ti-6Al-4V, commercial Ti-6Al-4V doped with BG, and optimal Ti-6Al-4V doped with BG two days after implantation. The three 3D-printed implants disclosed an unconnected distribution (shown in the red marked circle) of immature bone and mature bone at the bone–pedicle screw interface. However, more continuous distribution (shown in the blue marked circle) of mature bone was observed with the commercial Ti-6Al-4V doped with BG and optimal Ti-6Al-4V doped with BG than with the optimal Ti-6Al-4V after two days, which indicates the probable role of BG dopant in the improved direct osseous apposition. The sharp contrasts in 2D TXM images of spinal specimens depend on different absorption rates of the degree of bone calcification and only the pre-existing hard tissues of immature bone and mature bone were observed after two days for the three pedicle screw implants.

**Figure 5:**
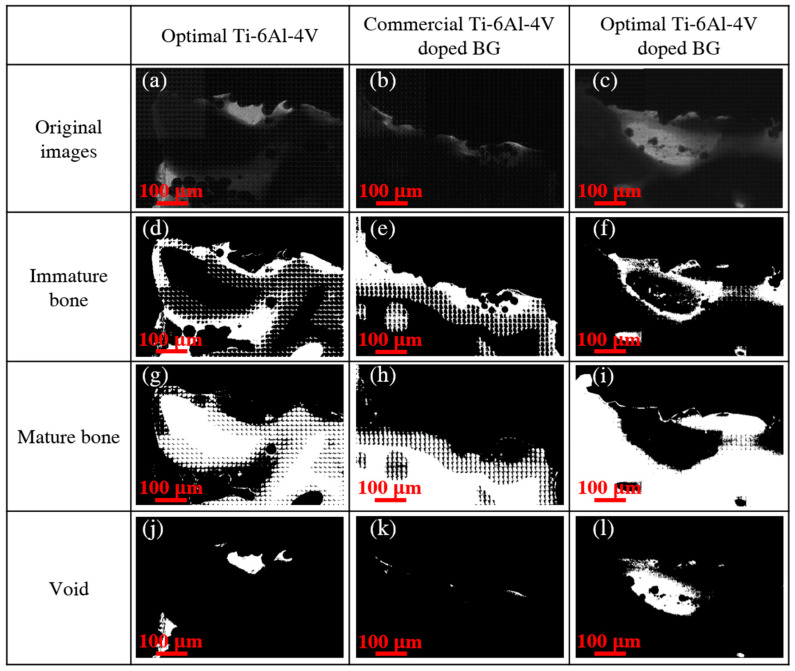
2D TXM images of bone remodeling around three 3D-printed pedicle screw implants of (**a**) optimal Ti-6Al-4V, (**b**) commercial Ti-6Al-4V doped with BG, and (**c**) optimal Ti-6Al-4V doped with BG 120 days after implantation. The white regions of immature bone, mature bone, and void are illustrated in (**d**), (**g**), and (**j**) for optimal Ti-6Al-4V; in (**e**), (**h**), and (**k**) for commercial Ti-6Al-4V doped with BG; and in (**f**), (**i**), and (**l**) for optimal Ti-6Al-4V doped with BG.

Nevertheless, with the increase in the implantation period to 120 days, a remarkable difference was observed regarding the remodeling status of immature bone and mature bone with the three pedicle screw implants, as illustrated in Figure 5. The development of mature bone rather than immature bone was more pronounced with coherent and continuous incorporation in all three 3D-printed implants, with the most prominent among them being the optimal Ti-6Al-4V doped with BG pedicle screw. Furthermore, since the void was observed 120 days after implantation but not 2 days after implantation, the appearance of void was presumably attributed to the evolution of soft tissues or cartilage during the implantation period. The combination of BG dopant and optimal shape design facilitated the development of mature bone, thereby enriching the surface osseointegration with the optimal Ti-6Al-4V doped with BG pedicle screw implant.

### 2.5. Most Successful Bone Remodeling with Optimal Ti-6Al-4V Doped with BG

**Figure 6:**
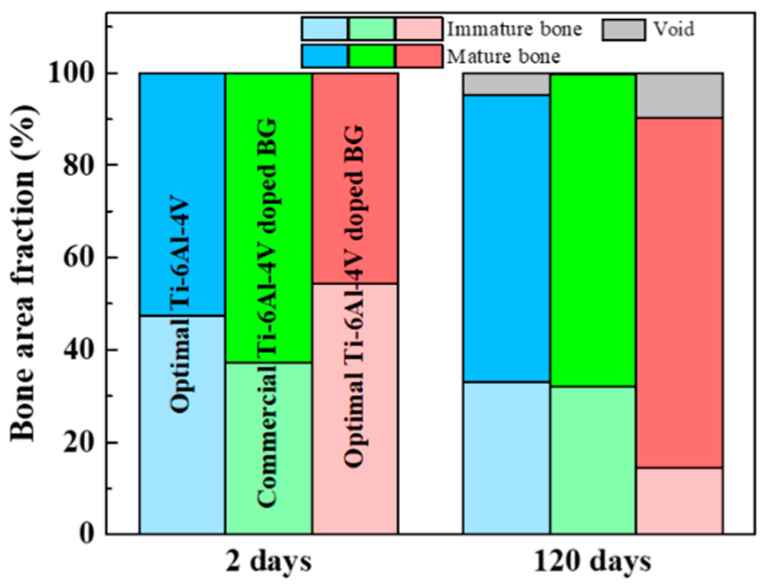
Area fraction of immature bone, mature bone, and void for the three 3D-printed pedicle screws of optimal Ti-6Al-4V, commercial Ti-6Al-4V doped with BG, and optimal Ti-6Al-4V doped with BG at 2 days and 120 days after implantation.

In terms of the biomechanical properties of healthy surrounding bone tissues, it is crucial to quantify the percentage of immature bone, mature bone, and void growth at the bone–implant interface. Figure 6 depicts the area fraction of immature bone, mature bone, and void in the three 3D-printed pedicle screws at 2 and 120 days after implantation.

Generally, a decrease in immature bone and an increase in developing mature bone accompanied by the appearance of void were observed for all three 3D-printed pedicle screws 120 days after implantation. Immature bone reduced from 47.5% to 33.0% and mature bone increased from 52.5% to 62.1% along with void of 4.9% for the optimal Ti-6Al-4V after 120 days. The commercial Ti-6Al-4V doped with BG exhibited a slight decrease in immature bone from 37.2% to 32.0% and an increase in mature bone from 62.8% to 67.7% after 120 days; however, a negligible presence of void of 0.3% was noted. Compared with the optimal Ti-6Al-4V and commercial Ti-6Al-4V doped with BG, the optimal Ti-6Al-4V doped with BG exhibited a more noticeable difference between the evolution of immature bone and mature bone during the same implantation period. The immature bone decreased from 54.4% to 14.4%, whereas a more striking development of mature bone from 45.6% to 75.8%, along with void of 9.8%, was obtained for the optimal Ti-6Al-4V doped with BG after 120 days. Notably, the BG dopant facilitates the increase of constituent HA minerals resulting in the rapid development of mature bone. During the same implantation period, the bone ingrowth process was mostly complete for the commercial shape-based implant, whereas the bone remodeling process seemed likely to continue in the optimal healing chamber-based pedicle screw. The synergistic combination of BG dopant and optimal shape design pedicle screw not only hastens the development of mineralized mature bone during the initial implantation period but also facilitates the subsequent gradual bone ingrowth for a complete bone healing process and successful long-term spinal stabilization.

## 3. Discussion

The success of pedicle screws in spinal fixation undergoes a complicated biomechanical process under various biophysical and mechanical stimuli, wherein the implants are initially stabilized, followed by the bone regeneration and bone incorporation around the implants, and finally, incorporation of the implants within the spinal structure as its part [41,42]. However, pedicle screw loosening is a common problem presented by the current screw design and material. If the optimal pedicle screw could act as a fusion material with long-lasting bone integration, it could potentially avoid the preparation of additional fusion areas and surgical interventions, such as posterolateral fusion or interbody fusion [43]. Nonetheless, excellent mechanical fixation and stabilization of pedicle screws require strong bone–implant interface bonding and direct biological anchorage to the surrounding bone tissue [44]. Notably, the first four months after implantation are crucial for evaluating the bone ingrowth and bone remodeling process around the implants after osseointegration.

The fracture healing process depends on the evolution of the surrounding bone tissue of fibrous tissue, cartilage, immature bone, and mature bone [42]. The formation of immature bone originates from the ossification of primarily constituent osteoblast cells, which regulate further nucleation and growth of HA mineral crystals [45]. Notably, the strength of the bone is gradually enhanced with time and is sufficiently strong to bear high loadings with the development of mineralized mature bone [46]. Therefore, superior bone ingrowth and greater bone maturity result in richer osseointegration, stronger bone–pedicle screw interface bonding, and faster fracture healing [8,28].

Our µCT results revealed that either the geometric effect of optimal shape design or the composition effect of the BG dopant had positive effects on the volume fraction of healthy surrounding bone; however, the effect of geometry on the bone volume fraction was more pronounced than the effect of the composition. Greater ingrowth of mineralized bone tissue into the well-designed healing chamber was attributed to a faster bone volume fraction in the optimal shape design pedicle screws than in the commercial shape ones. The combination of optimal shape design and BG dopant-based pedicle screw fabricated using 3D printing yielded the highest bone volume fraction and the highest amount of mature bone during the same implantation period, which strengthens the concept that bone–implant contact bonding fastens the bone remodeling process. Our superior results on the 3D-printed Ti-6Al-4V doped with BG pedicle screw with the optimal healing chamber suggested that new composite materials and optimal shape design should be applied simultaneously for creating potentially feasible implants for clinical applications that can accelerate the fracture healing process.

Nonetheless, this study had limitations. The number of spinal specimens examined using µCT were 2–4, whereas that investigated using TXM was one. Hence, a greater number of specimens in each sample set involved in each experiment is required to achieve statistical significance and excellent in vivo results that could help decide its feasibility for clinical applications. Furthermore, the mechanical pull-out tests, which are beneficial in directly evaluating the mechanical binding at the bone–pedicle screw interface, were not considered for discussion in the present study because of the incomplete results from an insufficient number of four types of pedicle screws. Notably, the current study focused on the developing status of bone integration and bone remodeling after spinal fusion within an essential implantation period of four months in vivo.

## 4. Materials and Methods

### 4.1. Preparation of Pedicle Screw Implants

**Figure 7:**
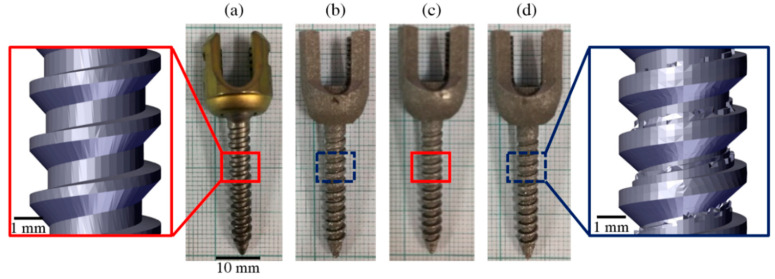
Photographs of four types of pedicle screws: (**a**) Commercial Ti-6Al-4V, (**b**) optimal Ti-6Al-4V, (**c**) commercial Ti-6Al-4V doped with BG, and (**d**) optimal Ti-6Al-4V doped with BG. Healing chambers of commercial and optimal shape-based pedicle screws are illustrated in the magnified images of Figure 7a,d, respectively.

Additive manufacturing was performed on an Industrial Technology Research Institute (ITRI)-AM 250 machine. Laser power of 170 W, scan speed of 1150 mm/s, hatch spacing of 0.1 mm, and laser spot size of 70 μm were used. The 3D printing of a commercial design-based pedicle screw composed of Ti-6Al-4V doped with 0.25% (expressed in weight percent) BG was fabricated using the SLM technique and compared with the commercially available Ti-6Al-4V pedicle screw, which was used as a reference implant to investigate the effect of the composition. In addition, the geometric effect was identified using two 3D-printed pedicle screws with an optimal shape design consisting of Ti-6Al-4V and Ti-6Al-4V doped with 0.25% BG. The BG used in this study was 58S with a melting point of 1400 °C, which was quite close to that of 1650 °C of Ti-6Al-4V. The 58S BG comprised 60 mol% SiO_2_, 36 mol% CaO, and 4 mol% P_2_O_5_ [39]. Our research findings indicated that 0.25% was the most appropriate concentration of BG for the additive manufacturing process, with the best mechanical properties. Photographs of four types of pedicle screw implants are illustrated in Figure 7. Notably, the healing chambers of commercial design-based pedicle screws have regular trapezoid shapes, whereas those of optimal shape design-based pedicle screws have a thicker bottom, as illustrated in the enlarged images of Figure 7a,d.

### 4.2. Preparation of Spine Specimens

A set of four types of pedicle screws and connecting rods [47], which provided in vivo stress, were surgically implanted in the thoracic and lumbar vertebrae of two healthy 1-year-old porcine animals (50 kg) from standard posterior approach. The pedicle screws were inserted under fluoroscopy guidance for confirmation of the final position. Some of the vertebrae were used explicitly for µCT, whereas some were prepared for traditional imaging techniques and TXM analyses. We assumed that all vertebrae could be compared with each other owing to the marginal differences between them. Animal experiments were performed strictly according to the regulations and laws of the Institutional Animal Care and Use Committee (IACUC) of Taipei Medical University (TMU), Taipei, Taiwan. All animal experiments were approved by the IACUC (IACUC number: LAC-2015-0281). The two porcine models were euthanized at pre-decided time points of 2 and 120 days after implantation following careful surgery, and the spinal specimens, including the pedicle screw implant and surrounding bone, were then fixed in formaldehyde and stored at −18 °C. The spinal specimens for imaging techniques, namely histological staining [48], fluorescence [49], µCT [50,51], and TXM, were prepared based on our previous protocol [28]. Histological staining, particularly RBS staining protocol proposed by Dorn and Hart Microedge, was employed for ground section histology. The spinal specimens were stained in acidified acid fuchsin for a clear observation of mature bone in the stained pink area under a transmitted light microscopy. Fluorescence microscopy technique was taken under an excitation wavelength of 467 nm and emission wavelength of 550 nm using a specific wavelength of the green filter (ET405/40x). Tetracycline was used as fluorochrome for the fluorescence characteristics. Fluorescence analysis allows an observation of immature bone in the brighter green fluorescence area.

### 4.3. Tensile Test

The in-situ neutron diffraction for tension was conducted using the VULCAN engineering diffractometer at the Spallation Neutron Source (SNS) of the Oak Ridge National Laboratory (ORNL). Neutron diffraction was used to characterize the structural properties, whereas the tensile test was performed to determine the mechanical properties of the 3D-printed Ti-6Al-4V and Ti-6Al-4V doped with BG specimens. The protocols of in-situ neutron diffraction for examination of the microstructure evolution of the tensile specimens are archived [52,53,54]. The tensile specimens of Ti-6Al-4V and Ti-6Al-4V doped with BG powders were horizontally built into dog-bone shapes by using the SLM technique with a gauge length of 10 mm and a gauge radius of 3 mm. The samples were strained at a strain rate of 1.2 × 10^−5^/min by using an extensometer for accurate strain measurement in the elastic deformation regime.

### 4.4. X-ray Micro-Computed Tomography

µCT was performed to calculate the volume of bone growth around the pedicle screws. Because of the image contrast in grayscale color and the penetration of X-ray through only the soft tissues of the living body [55], the hard bone and soft tissues can be distinguished as white and black regions, respectively. The spinal specimens were scanned using Skyscan 2211 at 25 μm. An ultra high voltage of 140 kV and a current of 155 μA were used for 360° scanning. Reconstructed cross section images were reorientated, and the region of interest was selected for further analysis. Overall, 150 image slices (corresponding to a thickness of 3.75 mm) were selected and considered as the total volume (TV) of the bone defect region. The distribution of bone in this region was calculated as the bone volume (BV). The ratio of BV to TV (BV/TV) was defined as the bone volume fraction.

### 4.5. Transmission X-ray Microscopy

We conducted TXM—a nondestructive image microscopy technique—at the wavelength of 1.54 Å (8 keV) with a spatial resolution of 60 nm at the beam line (BL) 01B1, National Synchrotron Radiation Research Center (NSRRC) in Taiwan. The evident contrasts of two-dimensional (2D) TXM images provided a clear differentiation of the developing areas of immature bone, mature bone, and void to the naked eye [28,56,57]. The surface area of bone ingrowth was determined as the ratio between the surface area of immature bone, mature bone, or void and the total area of bone ingrowth.

## 5. Conclusions

The stress-shielding effects can be effectively minimized by adding an optimal amount of 58S BG to 3D-printed Ti-6Al-4V alloy, which effectively lowers its elastic modulus of 92 GPa to be closer to that of bone. Compared with the optimal Ti-6Al-4V and commercial Ti-6Al-4V doped with BG pedicle screw implants, the optimal Ti-6Al-4V doped with BG resulted in superior bone ingrowth and long-lasting bone integration in vivo owing to its salient features of high bone volume fraction and mineralized mature bone. Our findings indicated that a combination of the optimal healing chamber and Ti-6Al-4V doped with BG pedicle screw fabricated through additive manufacturing facilitates osseointegration and accelerates bone healing and has the potential to be a promising implant for clinical applications.

## Abbreviations

Ti-6Al-4V	Titanium-aluminum-vanadium
BG	Bioactive glass
Ti	Titanium
HA	Hydroxyapatite
3D	Three-dimensional
SLM	Selective laser melting
µCT	X-ray micro-computed tomography
TXM	Transmission X-ray microscopy
S-S	Stress–strain
IACUC	Institutional Animal Care and Use Committee
TMU	Taipei Medical University
SNS	Spallation Neutron Source
ORNL	Oak Ridge National Laboratory
BL	Beamline
NSRRC	National Synchrotron Radiation Research Center
2D	Two-dimensional
